# Predicting factors and prediction model for discriminating between fungal infection and bacterial infection in severe microbial keratitis

**DOI:** 10.1371/journal.pone.0214076

**Published:** 2019-03-20

**Authors:** Passara Jongkhajornpong, Jirat Nimworaphan, Kaevalin Lekhanont, Varintorn Chuckpaiwong, Sasivimol Rattanasiri

**Affiliations:** 1 Department of Ophthalmology, Faculty of Medicine, Ramathibodi Hospital, Mahidol University, Bangkok, Thailand; 2 Department of Clinical Epidemiology and Biostatistics, Faculty of Medicine, Ramathibodi Hospital, Mahidol University, Bangkok, Thailand; University of California, UNITED STATES

## Abstract

A retrospective medical record review including 344 patients who were admitted with severe microbial keratitis at Ramathibodi Hospital, Bangkok, Thailand, from January 2010 to December 2016 was conducted. Causative organisms were identified in 136 patients based on positive culture results, pathological reports and confocal microscopy findings. Eighty-six eyes (63.24%) were bacterial keratitis, while 50 eyes (36.76%) were fungal keratitis. Demographics, clinical history, and clinical findings from slit-lamp examinations were collected. We found statistically significant differences between fungal and bacterial infections in terms of age, occupation, contact lens use, underlying ocular surface diseases, previous ocular surgery, referral status, and duration since onset (*p* < 0.05). For clinical features, depth of lesions, feathery edge, satellite lesions and presence of endothelial plaque were significantly higher in fungal infection compared to bacterial infection with odds ratios of 2.97 (95%CI 1.43–6.15), 3.92 (95%CI 1.62–9.45), 6.27 (95%CI 2.26–17.41) and 8.00 (95%CI 3.45–18.59), respectively. After multivariate analysis of all factors, there were 7 factors including occupation, history of trauma, duration since onset, depth of lesion, satellite lesions, endothelial plaque and stromal melting that showed statistical significance at *p* < 0.05. We constructed the prediction model based on these 7 identified factors. The model demonstrated a favorable receiver operating characteristic curve (ROC = 0.79, 95%CI 0.72–0.86) with correct classification, sensitivity and specificity of 81.48%, 70% and 88.24%, respectively at the optimal cut-off point. In conclusion, we propose potential prediction factors and prediction model as an adjunctive tool for clinicians to rapidly differentiate fungal infection from bacterial infection in severe microbial keratitis patients.

## Introduction

Microbial keratitis is one of the most common causes of corneal blindness across the globe, especially in the developing world [1, 2]. The estimated true incidence of microbial keratitis in South India was as high as 113 per 100,000 population [3] and its prevalence tends to rise each year [4]. The ratio of causative organisms varies from area to area [2]. Bacterial keratitis is most prevalent in most areas of the world, while fungal keratitis occupies a major portion of microbial keratitis in agriculture-based developing countries [2, 5]. Fungal keratitis has been known to be associated with more delayed diagnosis, longer admission period, and more expensive treatment with poorer results in visual outcome compared to bacterial keratitis [6–8]. Thus, an important key for improving treatment outcomes in fungal keratitis is to identify the cause of infection and begin proper treatment as soon as possible. This remains a major challenge for clinicians. Even for corneal specialists who diagnose patients based on clinical signs, the probability of correct pathogen differentiation between fungal and bacterial infection is less than 70% [9]. It is supposed to be even lower in the hands of general ophthalmologists or ophthalmology residents. In every presumed infectious keratitis case, a corneal specimen including microscopic examination and culture should be obtained for pathogen identification [9, 10]. The culture takes approximately 3 days for bacterial isolation and even up to 4 weeks for fungus cultures. Moreover, not uncommonly, these culture results appear negative regarding to an uncertain sensitivity of the test [2, 11]. An inaccurate diagnosis as well as delayed proper treatment can worsen clinical outcomes especially in patients with severe microbial keratitis [12], therefore efforts to find more rapid tests for pathogen identification have been in progress. Recently, new molecular techniques [13] and laser-scanning confocal microscopy [14, 15] have shown impressive results with high sensitivity and specificity for microbial isolation within a day. However, these tests require a degree of technology and experienced technicians to operate, thus they remain unavailable in most areas of the world [10]. Several studies focused on risk factors and clinical features that discriminate between bacteria and fungus [7, 16–19], but only few studies analyzed the sensitivity and specificity of using them as diagnostic tools [9, 20].

This study had 2 main purposes 1) to investigate the predicting factors that help discriminate between fungal infection and bacterial infection in patients with severe microbial keratitis and 2) to construct a prediction model for indicating fungal keratitis in patients with severe microbial keratitis. This study will provide clinicians with effective and rapid provisional diagnoses to help patients receive early appropriate treatment and consequently improve final visual outcome.

## Materials and methods

This 7-year retrospective study reviewed all admitted patients with severe microbial keratitis at Ramathibodi Hospital, a tertiary care center in Bangkok, Thailand, from January 2010 to December 2016. The study complied with the Declaration of Helsinki. The study protocol was approved and requirement for informed consent was waived by the Institutional Review Board of Ramathibodi Hospital.

Of 344 admitted patients with severe microbial keratitis, we excluded patients with incomplete medical records (6), unidentified pathogens (177), polymicrobial infection (12), pythium keratitis (7), presumed necrotizing herpes keratitis (3) and acanthamoeba keratitis (3). There were 136 patients for analysis. Severe microbial infection was determined by the presence of large corneal infiltrations (larger than 3 mm in the greatest diameter) and/or vision threatening corneal infiltrations which were located in 3-mm zone of corneal center with overlying epithelial defect.

### Data collection

Data were retrospectively extracted from medical records. In all subjects, demographics, referral status, duration since onset, local risk factors including contact lens use, ocular trauma and history of ocular surgeries, associated systemic disease and immunocompromised status were collected. Ocular findings from slit-lamp biomicroscopy including size, location, depth and specific clinical features were collected. Size was determined by the greatest diameter of the lesion measured by the length of the slit beam. Location was classified into 2 groups; central (lesions located within 3 mm from the corneal center) and peripheral (lesions more than 3 mm away from the corneal center). Depth of the ulcer was classified into 2 groups; anterior 2/3 of corneal thickness and posterior 1/3 of corneal thickness. The presence or absence of clinical features was noted, including feathery margin, satellite lesions, stromal necrosis, immune ring, generalized corneal haziness (ground glass appearance), endothelial plaque, and hypopyon in anterior chamber were noted.

All patients underwent corneal scraping by using a no.15 surgical blade with aseptic technique. Corneal specimens were collected from the base and active margin of the lesions. Then, samples were inoculated onto culture media consisting of blood agar, chocolate agar, thioglycollate broth and Sabouraud’s dextrose agar, and smeared on 2 glass slides for direct microscopic examination with Gram stained and 10% potassium hydroxide wet mount. The inoculated samples were incubated for 72 hours for bacterial isolations and 4 weeks for fungal isolations. The causative organism was considered if the same organism growth at the site of inoculation on two or more solid media, or growth at site of inoculation on one solid media of an organism consistent with microscopic finding, or confluent growth on one media was observed [21]. For negative-cultured cases, causative organisms were identified as fungi or bacteria based on positive findings from confocal microscopy or from corneal tissue pathology reported from an experienced pathologist. The corneal tissue sections were routinely stained with hematoxylin and eosin (H&E) and periodic acid Schiff stain (PAS), then if the corneal tissue revealed acute suppurative keratitis or acute necrotizing keratitis, the appropriate special stains including Gomori methenamine stain (GMS), Brown Brenn stain and 1% acid fast stain were performed for further organism identification. Confocal microscopic examination was done by using a Nidek ConfoScan 4 (Albignasego, Italy) with a Zeiss Achroplan ×40 lens. The presence of highly reflective, septate, double-walled filaments sizing between 3–8 microns was considered as fungal keratitis [15]. The results of confocal microscopy were reviewed and defined by a single experienced cornea specialist (KL).

### Statistical analysis

Data analysis was performed using STATA software, version 15 (StataCorp 2011, College Station, TX, USA). To describe the samples, mean and SDs were used for continuous variables and frequency and percentages for categorical variables. We compared the differences of historical factors and ocular features between fungal and bacterial keratitis by using the Chi-square test (or Fisher exact test). Odds ratios (OR) were estimated by using a simple logistic regression. Factors that were significant at *p* < 0.10 in univariate analysis were considered for multivariate analysis. Multiple logistic regression was used to predict factors associated with fungal keratitis. *P* values < 0.05 were considered statistically significant. The likelihood ratio (LR) test with backward elimination procedure was used to select the best model. The area under receiver operating characteristic (ROC) curve was estimated to distinguish fungal infection from bacterial infection with consideration of all significant factors from multivariate analysis. Logistic regression coefficients were used to create a scoring scheme. All subjects were allocated a coefficient according to their risk factors and then summed to get the total score. The cut-off value for classifying patients with high risk or low risk of fungal infection was selected based on the value of likelihood ratios, sensitivity and specificity. Sensitivity is the ability of the model to correctly identify cases with fungal keratitis (true positive), whereas the specificity is the ability of the model to correctly identify cases with bacterial keratitis (true negative).

## Results

Of a total of 136 severe microbial keratitis cases, there were 86 patients (63.24%) with bacterial keratitis and 50 patients (36.76%) with fungal keratitis. One hundred and thirteen cases (83.09%) were culture-positive (32 patients with fungal keratitis and 81 patients with bacterial keratitis) as shown in Table 1. Meanwhile, 23 culture-negative cases (16.91%) were diagnosed by confocal microscopy in 13 patients (9.56%) and corneal tissue pathology in 10 patients (7.35%). All cases diagnosed by confocal microscopy were fungal keratitis and all of the diagnoses were confirmed by successful treatment with anti-fungal medications.

**Table 1:** Causative organisms and relative frequency.

Organisms (number)	Number (n = 113)	Percentage (%)
**Bacteria (81)**		
Gram-positive cocci (11)		
*Staphylococcus spp*.		
• coagulase negative	3	2.65
• coagulase positive	4	3.54
*Streptococcus pneumoniae*	4	3.54
Gram-positive rod (6)		
*Propionibacterium acnes*	6	5.31
Gram-negative bacilli (61)		
*Pseudomonas spp*.		
• *P*. *aeruginosa*	53	46.9
• *P*. *otitidis*	1	0.88
*Citrobacter spp*.	1	0.88
*Morganella morganii*	2	1.77
*Moraxella lacunata*	1	0.88
*Proteus Mirabilis*	1	0.88
*Serratia marcescens*	1	0.88
*Stenotrophomonas maltophilia*	1	0.88
*Mycobaterium abscessus*	3	2.65
**Fungus (32)**		
Hyaline fungi (29)		
*Fusarium spp*.	7	6.19
*Aspergillus spp*.	6	5.31
*Lasiodiplodia spp*.	2	1.77
*Bipolaris spp*.	2	1.77
*Botryosphaeria spp*.	2	1.77
*Acremonium spp*.	3	2.65
*Diaporthe phaseolorum*	1	0.88
*Colletotrichum spp*.	2	1.77
*Neodeightonia subglobosa*	1	0.88
*Ramularia spp*.	1	0.88
Non-sporulated fungi	2	1.77
Dematiaceous fungi (3)		
*Curvularia spp*.	3	2.65

### Historical factors

The median age was 58 years (ranged 2–87 years). There were more females than males (52.21% vs 47.79%). Few patients had agricultural occupations (13.97%), 14.71% had diabetes, and 17.65% were contact lens use. Approximately one third of the patients had underlying ocular surface diseases prior to onset of keratitis (32.35%), history of ocular surgeries (31.62%) and history of trauma (30.88%) (Table 2). Over a half of the patients were referred from other hospitals (55.15%) and had long duration since onset of more than 3 days (61.76%) as shown in Table 2. The median duration was 4 days (ranged 1 to 180 days).

**Table 2:** Univariate analysis of historical data and clinical features comparing between fungus and bacterial keratitis.

Factors	Fungal Keratitis	Bacterial Keratitis	OR*	95%CI	P value
	n = 50 (%)	n = 86 (%)			
**Historical factors**					
Age					
> 40 years	45 (90)	56 (65.12)	4.82	1.73, 13.44	0.003**
≤ 40 years	5 (10)	30 (34.88)	1		
Gender					
male	27 (54)	38 (44.19)	1.48	0.74, 2.99	0.27
female	23 (46)	48 (55.81)	1		
Occupation					
agricultural	17 (34)	2 (2.33)	21.64	4.73, 98.88	<0.001**
non-agricultural	33 (66)	84 (97.67)	1		
Diabetes					
yes	6 (12)	14 (16.28)	0.5	0.25, 1.96	0.498
no	44 (88)	72 (83.72)	1		
Contact lens use					
yes	0 (0)	24 (27.91)	3.88x10^-3^	0, 0.07	<0.001**
no	50 (100)	62 (72.09)	1		
History of ocular surface diseases					
Yes	8 (16)	36 (41.86)	0.26	0.11, 0.63	0.003**
no	42 (84)	50 (58.14)	1		
History of ocular surgery					
penetrating keratoplasty	4 (8)	20 (23.26)	0.28	0.09, 0.87	0.029**
non-penetrating keratoplasty	7 (14)	12 (13.95)	0.81	0.29, 2.24	0.681
no	39 (78)	54 (62.74)	1		
History of trauma					
agricultural	15 (30)	4 (4.65)	13.04	3.91, 43.50	<0.001**
non-agricultural	14 (28)	9 (10.47)	5.41	2.05, 14.23	0.001**
no	21 (42)	73 (84.88)	1		
Referral status					
yes	35 (70)	40 (46.51)	2.68	1.28, 5.62	0.009**
no	15 (30)	46 (53.49)	1		
Duration since onset					
> 3 days	45 (90)	39 (45.35)	10.62	3.84, 29.37	<0.001**
≤ 3 days	5 (10)	47 (54.65)	1		
**Clinical features**					
Size					
> 3 mm	45 (90)	47 (54.65)	1.2	0.57, 2.51	0.633
≤ 3 mm	5 (10)	39 (45.35)	1		
Depth					
posterior stroma	33 (66)	34 (66)	2.97	1.43, 6.15	0.003*
anterior to mid stroma	17 (34)	52 (60.47)	1		
Location					
central	33 (66)	50 (58.14)	1.4	0.68, 2.89	0.366
non-central	17 (34)	36 (41.86)	1		
Feathery edge					
yes	17 (34)	10 (11.63)	3.92	1.62, 9.45	0.002*
no	33 (66)	76 (88.37)	1		
Satellite lesions					
yes	16 (32)	6 (6.98)	6.27	2.26, 17.41	<0.001*
no	34 (68)	80 (93.02)	1		
Multifocal lesions					
yes	3 (6)	3 (3.49)	1.77	0.34, 9.10	0.497
no	47 (94)	83 (96.51)	1		
Ring infiltration					
yes	2 (4)	7 (8.14)	0.47	0.94, 2.36	0.359
no	48 (96)	79 (91.86)	1		
Stromal melting					
yes	15 (30)	47 (54.65)	0.36	0.17, 0.75	0.006*
no	35 (70)	39 (45.35)	1		
Ground glass appearance					
yes	1 (2)	19 (22.09)	0.07	0.01, 0.56	0.012*
no	49 (98)	67 (77.91)	1		
Pigmentation					
yes	2 (4)	0 (0)	2.98x10^-4^	0, 0.01	0.133
no	48 (96)	86 (100)	1		
Hypopyon					
> 1 mm	11 (22)	24 (27.91)	1.53	0.68, 3.45	0.301
≤ 1 mm	23 (46)	30 (34.88)	0.92	0.36, 2.33	0.855
no	16 (32)	32 (37.21)	1		
Endothelial plaque					
yes	27 (54)	11 (12.79)	8	3.45, 18.59	<0.001*
no	23 (46)	75 (87.21)	1		

OR: odds ratio, CI: confidence interval

* odds ratios were analyzed by using simple logistic regression (code 1 for fungal keratitis and code 0 for bacterial keratitis).

** indicates statistical significance at *p* < 0.05.

In univariate analysis, age, occupation, contact lens use, underlying ocular surface diseases, history of ocular surgery, history of trauma, referral and duration since onset before admission significantly differed between fungal and bacterial keratitis as demonstrated in Table 2. Older age (> 40 years), agricultural occupation, history of trauma, referral and long duration since onset (> 3 days) was significantly associated with fungal keratitis, while underlying ocular surface diseases and history of penetrating keratoplasty were significantly higher in bacterial keratitis. From multivariate analyses, only 3 historical data categories consisting of agricultural occupation, history of trauma from agricultural foreign bodies and long duration since onset showed statistical significance (Table 3).

**Table 3:** Multivariate analysis of historical data and clinical features comparing fungus and bacterial keratitis.

Factors	OR	95%CI	P value
**Historical factors**			
Occupation			
agricultural	8.33	1.55, 44.89	0.014
non-agricultural	1		
Trauma			
agricultural	4.6	1.05, 20.14	0.043
non-agricultural	2.67	0.92, 7.76	0.072
no	1		
Duration since onset			
> 3 days	7.8	2.52, 24.12	<0.001
≤ 3 days	1		
**Clinical features**			
Depth			
posterior stroma	4.08	1.61, 10.35	0.003
anterior to mid stroma	1		
Satellite lesions			
yes	5.03	1.44, 17.61	0.012
no	1		
Endothelial plaque			
yes	5.63	2.19, 14.49	<0.001
no	1		
Stromal melting			
yes	0.27	0.11, 0.69	0.006
no	1		

OR: odds ratio, CI: confidence interval

### Clinical features

Overall, 61.30% lesions were centrally located and 50% lesions involved the posterior stroma. The mean size of lesions was 4.2 ± 2.1 mm. Regarding the specific clinical features, endothelial plaque was the most prevalent finding in patients with fungal infection (54%) followed by feathery edge (34%) and satellite lesions (32%). Whereas stromal melting was the most common feature found in bacterial infection followed by hypopyon (27.91%) and ground glass appearance (22.09%) as demonstrated in Table 2.

In univariate analysis, depth of lesions, specific signs including feathery edge, satellite lesions, stromal melting, ground glass appearance and endothelial plaque showed a statistically significant difference between the 2 groups (Table 2). Lesions involving posterior stroma (OR 2.97, 95%CI 1.43–6.15), feathery edge (OR 3.92, 95%CI 1.62–9.45), satellite lesions (OR 6.27, 95%CI 2.26–17.41), and endothelial plaque (OR 8.00, 95%CI 3.45–18.59) were found significantly higher in fungal infection compared to bacterial infection. While stromal melting (OR 0.36, 95%CI 0.17–0.75) and ground glass appearance (OR 0.07, 95%CI 0.01–0.56) were found to appear significantly less frequently in patients with fungal keratitis compared to bacterial keratitis. After multivariate analysis, 4 clinical features including depth of lesions, satellite lesions, endothelial plaque and stromal melting showed statistical significance (Table 3).

### Prediction model

We constructed a prediction model to differentiate between fungal keratitis and bacterial keratitis based on 7 factors including 3 historical and 4 clinical features by using coefficient values from multivariate analyses (Table 4). The model showed good sensitivity, specificity and correct classification at 70.00%, 88.24% and 81.48%, respectively (Table 5). By using the receiver operating characteristic (ROC) curve analysis, the ROC area was 0.79 (95%CI 0.72–0.86).

**Table 4:** Scoring scheme using coefficient values.

Model parameters	Coefficient values
Intercept	-2.989
**Historical factors**	
Trauma	
agricultural	5.358
non-agricultural	1.509
no	0
Duration since onset	
> 3 days	2.206
≤ 3 days	0
**Clinical features**	
Endothelial plaque	
yes	2.159
no	0
Stromal melting	
yes	-1.121
no	0

**Table 5:** The sensitivity, specificity, positive likelihood ratio, correct classification, and area under receiver operating characteristic (ROC) at the optimal cut-off point (0.25).

Cut off point	Sensitivity	Specificity	Positive likelihood ratio	Correct classification	ROC
0.25	70.00	88.24	5.95	81.48	0.79

## Discussion

This current study demonstrated the critical role of historical data as well as observed clinical signs in helping clinicians to discriminate causative organisms at the time of presentation with favorable accuracy, sensitivity and specificity. Although slide smear and cultures from corneal scraping are considered the gold standard investigation for microbial keratitis, there is a recovery rate of less than 50% for the tests in several studies [6, 22–25]. Furthermore, microbiological tests are still limited in many parts of developing countries due to lack of facilities, equipment and expert laboratory staff. Even in developed countries with comprehensive labs, not every case with corneal ulcer is scraped for organism identification [23, 26].

### Historical factors

Similar to previous studies, compared with bacterial keratitis, fungal infection was more likely to occur in older patients, agricultural occupations, ocular trauma especially with agricultural foreign bodies, referral patients and patients with longer duration since onset [7, 19]. Contact lens use has been known as an important risk factor for bacterial keratitis, especially pseudomonas keratitis, but less likely to be associated with fungal keratitis [7]. Only one unusual global outbreak of fusarium keratitis in contact lens use occurred during 2005–2006, which was possibly associated with alexidine composited in ReNu with MoistureLoc contact lens solution [27]. In this study, we included a small portion of contact lens use (24 eyes, 17.65%). Similar to previous studies [28, 18], no contact lens use had fungal keratitis. Although the history of contact lens use showed strong negative association with fungal infection, we were unable to analyze it in multivariate analysis as well as in the prediction model because of zero frequency in fungal group. Our result supports the findings from previous studies that pre-existing ocular surface diseases and previous ocular surgeries particularly penetrating keratoplasty (PKP) showed significant correlation with bacterial keratitis [18, 29].

### Clinical features

From previous study, approximately 63% of patients with fungal keratitis was successfully diagnosed based on clinical signs and symptoms [30]. Another study from Dahlgren MA et al found that only 42% of the diagnoses made by using clinical features were correct [17]. Furthermore, clinicians using clinical features had lower probability of making correct diagnoses for fungal keratitis compared to bacterial keratitis (62% and 69%, respectively) [9]. Size and location of the lesions revealed no significant association with causative pathogens, however they are important parameters in terms of treatment planning. Deeper infiltrations are more likely to occur in eyes with fungal keratitis. The most common feature found in fungal infections was endothelial plaque followed by feathery edge and satellite lesions. Feathery edge is universally reported as a sign of fungal infection [9, 23, 31]. Ring infiltration was considered as a non-specific sign, though it possibly indicates a long disease duration [5]. Similar to immune ring, multifocal lesions and the presence of hypopyon were observed in both fungal and bacterial keratitis at a comparable proportion, therefore we considered them as non-specific signs. Furthermore, there were no significant differences in the levels of hypopyon between bacterial and fungal infection. It should be noted that the definitions of satellite lesions and multifocal lesions have not been clearly described and might vary from observer to observer. However, from our experience, the disproportion between a level of hypopyon and the size of lesions might be an important clue for presumptive diagnosis of fungal keratitis. Only one study from Bangladesh showed a significantly higher level of hypopyon found in Pneumococcal ulcers compared to that found in Pseudomonas ulcers [31]. Interestingly, endothelial plaque is strongly associated with fungal infection with the highest odds ratio of 8.00, corresponding with the finding from Dunlop AA et al [31]. Pigmentation was observed in only 2 cases infected from *Curvularia spp*. and another unidentified fungus. Evidence from previous reports and our study confirmed that the presence of pigmentation in the lesion strongly suggests fungal infection [23, 31]. Because of a rare presence of pigmentation, it showed an insignificant association in statistical analysis and we were unable to include this sign into the prediction model analysis. As expected, ground glass appearance and stromal melting were two important signs indicating bacterial infection.

### Prediction model

In terms of diagnostic score, Thomas PA et al demonstrated that the probability of fungal infection was 63% if 1 of 3 clinical features including a serrated margin, raise slough and coloration, was detected [23]. If all clinical features presented, the probability of fungal infection would increase to 83% [23]. Because of the few clinical features recruited in the model and the score having not been weighed according to the odds ratios or coefficient values, their proposed model showed a large gap of sensitivity and specificity between each score. It has been discussed that the duration of symptoms sometimes seems unreliable and imprecise especially in patients with a long history [23], thus in this study, we broadly classified patients into 2 groups at the cut point of 3 days to minimize errors. The ROC analysis indicated that the final model showed favorable likelihood ratios, sensitivity and specificity at the cut-off point of 0.25 (S1 Table). In clinical practice, determining the initial treatment is not only based on clinical history and ocular findings. Proper diagnosis also depends on the prevalence of pathogens and medical availability in each area. Adjustment of the cut-off point is required in areas where fungal infection prevalence is largely different from our profile. Raising the cut-off point is recommended when using the model in temperate areas where a low incidence of fungal infection is reported.

Our limitations included some flaws related to the nature of a retrospective-designed study. First, data were obtained from medical records by several evaluators consisting of ophthalmology residents, fellows and cornea specialists. Underestimation of various clinical features could have occurred due to less experience and training by our residents, however, since the numbers were low, they did not have a significant effect on overall results. Second, Thailand is a developing country and located in a tropical zone, our patients had their own region-specific characteristics and had a high prevalence of fungal keratitis therefore cut-off point adjustment should be considered before applying to different conditions. Third, our predictive scores based on coefficient values which was differ from the previous score that was simplified into the round-numbered score [23]. Total score in our model derived from the summation of intercept and coefficients of each factors in Table 4. This might take time for clinicians to score and apply the results to individual patients. Despite these limitations, we believe that our approach will provide the most accurate predictive model for pathogen discrimination between bacterial and fungal infection based on historical and clinical data for patients with severe microbial keratitis.

Treatment guidelines for microbial keratitis generally indicate empirical treatment with broad-spectrum antibiotics covering gram positive and gram negative bacteria [32]. However, in developing countries or areas with a high prevalence of fungal infection, this approach might not be applicable. Using our predictive score, patients that score higher than the 0.25 cut-off value are likely to have infection caused by fungus. Therefore, treatment with anti-fungal medication alone or combination with broad-spectrum antibiotics is recommended.

## Conclusion

We identified 7 predictive factors and constructed a prediction model to differentiate between fungal and bacterial infection based on both historical and clinical data. Our predictive tool helps clinicians promptly choose appropriate investigation and treatment which ultimately improves treatment outcomes inpatient with severe microbial keratitis. This model can be applied in every hospital service levels, from primary care centers where lack of laboratory resource to referral care centers where laboratory tests take time.

## Supporting information

S1 Table Sensitivity, specificity, correctly classified, positive and negative likelihood ratios of each cut-off point. (DOCX)

S1 Dataset Clinical data of all patients. (XLS)
