# Supplementary material for: Predicting factors and prediction model for discriminating between fungal infection and bacterial infection in severe microbial keratitis
Source: PLoS One. 2019 Mar 20;14(3):e0214076. doi: 10.1371/journal.pone.0214076 (PMC6426210; doi:10.1371/journal.pone.0214076)
Supplement: S1 Table — (DOCX) [file pone.0214076.s001.docx]

**S1Table.** Sensitivity, specificity, correctly classified, positive and negative likelihood ratios of each cut-off point.

| Cut-off point | Sensitivity | Specificity | Correctly Classified | LR+ | LR- |
| --- | --- | --- | --- | --- | --- |
| -4.11 | 100.00% | 0.00% | 37.04% | 1 | - |
| -2.99 | 100.00% | 27.06% | 54.07% | 1.371 | 0 |
| -1.95 | 100.00% | 45.88% | 65.93% | 1.8478 | 0 |
| -1.9 | 100.00% | 50.59% | 68.89% | 2.0238 | 0 |
| -1.48 | 92.00% | 61.18% | 72.59% | 2.3697 | 0.1308 |
| -0.83 | 90.00% | 61.18% | 71.85% | 2.3182 | 0.1635 |
| -0.78 | 88.00% | 62.35% | 71.85% | 2.3375 | 0.1925 |
| -0.63 | 78.00% | 80.00% | 79.26% | 3.9 | 0.275 |
| -0.44 | 76.00% | 81.18% | 79.26% | 4.0375 | 0.2957 |
| -0.39 | 76.00% | 82.35% | 80.00% | 4.3067 | 0.2914 |
| 0.25 | 70.00% | 88.24% | 81.48% | 5.95 | 0.34 |
| 0.45 | 64.00% | 90.59% | 80.74% | 6.8 | 0.3974 |
| 0.68 | 60.00% | 92.94% | 80.74% | 8.5 | 0.4304 |
| 0.73 | 58.00% | 92.94% | 80.00% | 8.2167 | 0.4519 |
| 1.38 | 50.00% | 95.29% | 78.52% | 10.625 | 0.5247 |
| 1.53 | 34.00% | 97.65% | 74.07% | 14.45 | 0.6759 |
| 1.57 | 32.00% | 97.65% | 73.33% | 13.6 | 0.6964 |
| 1.76 | 26.00% | 98.82% | 71.85% | 22.1001 | 0.7488 |
| 2.61 | 24.00% | 100.00% | 71.85% | - | 0.76 |
| 2.88 | 20.00% | 100.00% | 70.37% | - | 0.8 |
| 3.73 | 12.00% | 100.00% | 67.41% | - | 0.88 |
| 3.73 | 0.00% | 100.00% | 62.96% | - | 1 |
